# Pancreatic stellate cells: a starring role in normal and diseased pancreas

**DOI:** 10.3389/fphys.2012.00344

**Published:** 2012-08-28

**Authors:** Minoti V. Apte, Romano C. Pirola, Jeremy S. Wilson

**Affiliations:** Pancreatic Research Group, Faculty of Medicine, South Western Sydney Clinical School, University of New South WalesSydney, NSW, Australia

**Keywords:** pancreatic fibrosis, stellate cells, chronic pancreatic, desmoplastic reaction, pancreatic cancer, review

## Abstract

While the morphology and function of cells of the exocrine and endocrine pancreas have been studied over several centuries, one important cell type in the gland, the pancreatic stellate cell (PSC), had remained undiscovered until as recently as 20 years ago. Even after its first description in 1982, it was to be another 16 years before its biology could begin to be studied, because it was only in 1998 that methods were developed to isolate and culture PSCs from rodent and human pancreas. PSCs are now known to play a critical role in pancreatic fibrosis, a consistent histological feature of two major diseases of the pancreas—chronic pancreatitis and pancreatic cancer. In health, PSCs maintain normal tissue architecture via regulation of the synthesis and degradation of extracellular matrix (ECM) proteins. Recent studies have also implied other functions for PSCs as progenitor cells, immune cells or intermediaries in exocrine pancreatic secretion in humans. During pancreatic injury, PSCs transform from their quiescent phase into an activated, myofibroblast-like phenotype that secretes excessive amounts of ECM proteins leading to the fibrosis of chronic pancreatitis and pancreatic cancer. An ever increasing number of factors that stimulate and/or inhibit PSC activation via paracrine and autocrine pathways are being identified and characterized. It is also now established that PSCs interact closely with pancreatic cancer cells to facilitate cancer progression. Based on these findings, several therapeutic strategies have been examined in experimental models of chronic pancreatitis as well as pancreatic cancer, in a bid to inhibit/retard PSC activation and thereby alleviate chronic pancreatitis or reduce tumor growth in pancreatic cancer. The challenge that remains is to translate these pre-clinical developments into clinically applicable treatments for patients with chronic pancreatitis and pancreatic cancer.

## Introduction

“I will love the light for it shows me the way, yet I will endure the darkness because it shows me the stars.” Og Mandino (American Essayist and Psychologist, 1923–1996).

Although both exocrine and endocrine functions of the pancreas and the cell types relevant to these functions (acinar cells, ductal cells, and cells of the islets of Langerhans) have been extensively studied since the pancreas was first described by Herophelus (335–280 BC) (Howard and Hess, 2002), one major cell type remained in the dark until as recently as 20 years ago. This cell type, the pancreatic stellate cell (PSC, so called because of its star-like appearance *in situ*) was first reported by Watari (Watari et al., 1982) in 1982 using electron microscopy of rodent and human pancreas. Watari likened these cells to hepatic stellate cells, which are well established as the key effector cells in liver fibrosis. However, there was little further effort in the field to characterize PSCs or to determine whether they played a similar fibrogenic role in the pancreas as their hepatic counterparts. This possibly reflected a general disinterest at the time in the mechanisms of pancreatic fibrosis which, despite being a predominant histological feature of two major pancreatic diseases (chronic pancreatitis and pancreatic cancer), was mostly considered to be an epiphenomenon of chronic injury.

It was to be another 16 years since Watari's initial report before methods were developed to isolate and culture PSCs, which finally provided researchers with a tool to study the mechanisms responsible for pancreatic fibrosis. In general, fibrosis is defined as the excessive accumulation of extracellular matrix (ECM) proteins (particularly fibrillar collagens) as a result of a loss of the normal balance between the deposition and the degradation of ECM. The concept of fibrosis as an inert, reactive tissue has changed significantly in recent times. Indeed, it is now well recognized that fibrogenesis in the pancreas is an active, dynamic process that may be reversible, at least in the early stages. Importantly, there is unequivocal evidence to indicate that PSCs play a central role in pancreatic fibrogenesis (Apte et al., 2011).

## The history of stellate cells

In contrast to the pancreas, where the fibrogenic process has only received attention in recent years, the mechanisms of fibrosis in the liver have been well studied over several decades. Stellate cells were identified in the liver more than 130 years ago by the famous pathologist Karl Wilhelm von Kupffer. In a letter written in 1876 to his colleague Heinrich von Waldeyer, Kupffer described star shaped cells (“sternzellen”) stained with gold chloride in perisinusoidal spaces in the liver. However, Kupffer was unsure at the time as to whether these cells were different from resident liver macrophages, and this resulted in considerable confusion in the field for several decades. Almost 75 years after Kupffer's first description of “sternzellen,” Ito (Ito, 1951) reported the presence of lipid-containing cells in a perisinusoidal location in the liver which he termed as Ito cells. Finally in 1971, Wake and colleagues (Wake et al., 1987), used multiple techniques (gold chloride staining, lipid staining and electron microscopy) which clearly demonstrated that vitamin A storing hepatic stellate cells were the same as the Ito cells and also the same as the sternzellen initially reported by Kupffer, but were distinctly different from liver macrophages (also known as Kupffer cells). Rapid progress was made in the field following this clarification, with detailed characterization of the biology and functions of hepatic stellate cells, from the first report of the possible role of HSCs in collagen synthesis by Kent and colleagues (Kent et al., 1976) in 1976, to the current time where HSCs are established as not only central to the production of ECM proteins but also as serving several other functions including roles in liver development and regeneration, retinoid metabolism and immunomodulation (Lee and Friedman, 2011).

As noted earlier, the pancreatic counterparts of HSCs were first described by Watari in 1982, (more than a hundred years after Kupffer's initial reports in the liver). Watari examined the pancreas of vitamin A loaded mice by fluorescence microscopy and electron microscopy and described the presence of cells exhibiting a rapidly fading blue-green fluorescence characteristic of vitamin A in the periacinar areas of the pancreas (Watari et al., 1982). In 1990, a similar (albeit sparsely distributed) vitamin A autofluorescence was reported in normal pancreatic sections from rats and humans by Ikejiri (Ikejiri, 1990). A few years later, in 1998, two seminal papers were published describing the isolation and culture of PSCs from rat and human pancreas (Apte et al., 1998; Bachem et al., 1998). These methods proved to be a major breakthrough because they finally provided an invaluable *in vitro* tool for researchers to characterize the biology of PSCs in health and disease.

## Pancreatic stellate cells

**Figure 1:**
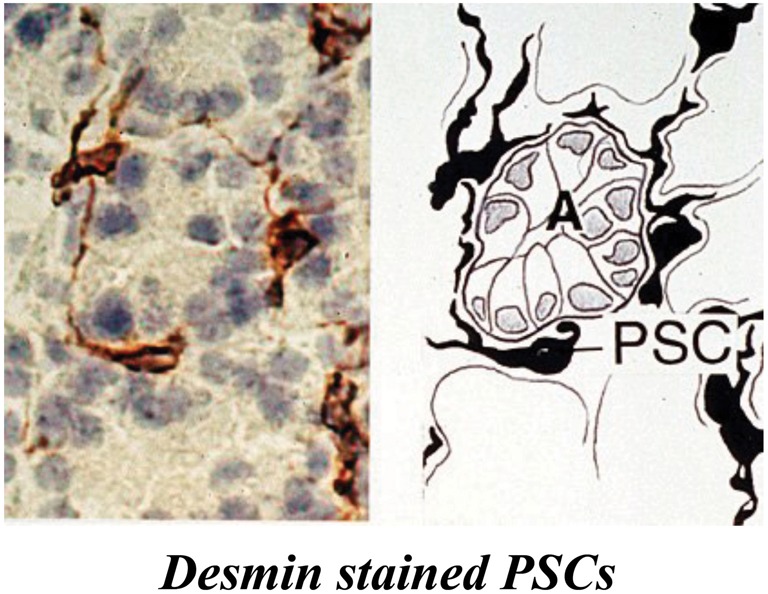
**Pancreatic stellate cells in rat pancreas stained for the selective marker desmin.** The left panel shows a representative photomicrograph of normal rat pancreas immunostained for desmin. The right panel depicts the corresponding line diagram. Desmin positive PSCs with long cytoplasmic projections are located at the basolateral aspect of acinar cells (A). *Reprinted with permission from BMJ Group*.

PSCs are located adjacent to the basolateral aspects of pancreatic acinar cells and have also been identified around small pancreatic ducts and blood vessels (Figure 1) (Watari et al., 1982; Ikejiri, 1990; Apte et al., 1998). They comprise approximately 4–7% of the total cell mass in the gland (Apte et al., 1998; Bachem et al., 1998). In the healthy pancreas, PSCs exhibit abundant, vitamin A containing lipid droplets in their cytoplasm and are in their quiescent (non-activated) state. They can be differentiated from fibroblasts due to their expression of selective markers such as desmin, glial fibrillary acidic protein (GFAP), vimentin and nestin (intermediate filament proteins) and neuroectodermal markers such as nerve growth factor (NGF) and neural cell adhesion molecule (NCAM). On electron microscopic examination, PSCs reveal a prominent rough endoplasmic reticulum, collagen fibrils and vacuoles (lipid droplets) surrounding a central nucleus.

### Characteristics of quiescent (non-activated) PSCs

**Figure 2:**
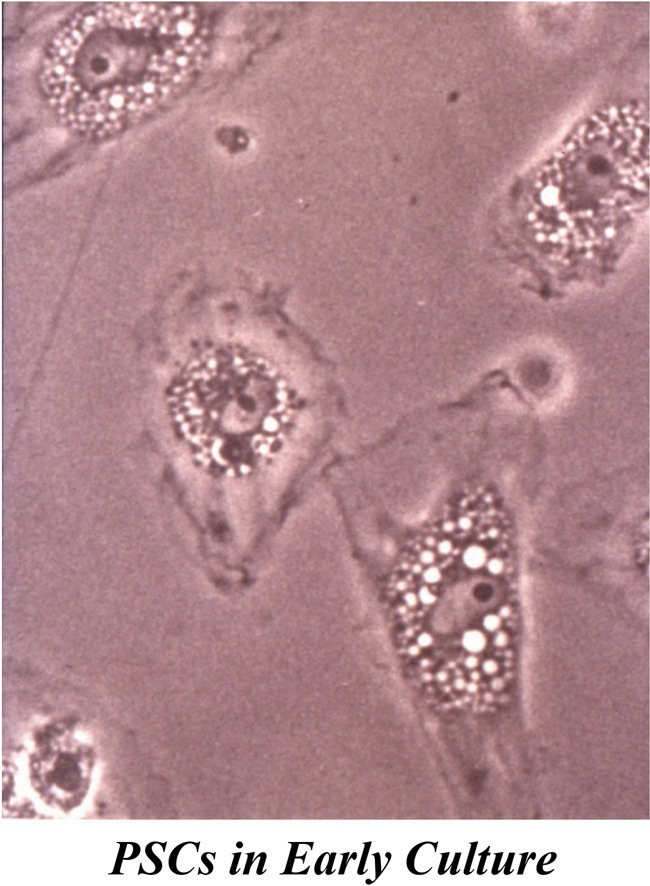
**PSCs in early culture.** The cells exhibit a flattened polygonal shape with abundant lipid droplets (containing vitamin A) in the cytoplasm, surrounding the central nucleus. *Reprinted with permission from BMJ Group.*

These have been essentially determined using PSCs isolated from normal rat and human pancreas. Taking their cues from the method used to isolate HSCs, Apte et al. (1998) developed a technique to isolate PSCs based on the knowledge that in the normal pancreas, PSCs contain abundant lipid droplets in their cytoplasm which decreases cell density. Consequently, when a suspension of pancreatic cells is centrifuged through a density gradient, PSCs can be readily separated from other pancreatic cells (Apte et al., 1998). When placed in plastic culture wells, quiescent PSCs exhibit a flattened polygonal shape with prominent lipid droplets in the cytoplasm surrounding the central nucleus (Figure 2). Exposure of the cells to UV light at 328 nm elicits a transient blue-green fluorescence typical of vitamin A. After being in culture for a period of about 48 h, these quiescent PSCs become activated, a process that is inevitably associated with a loss of the cytoplasmic vitamin A droplets and a transformation of cell shape to a myofibroblast like phenotype that now expresses the cytoskeletal protein α smooth muscle actin (αSMA).

Although the presence of vitamin A containing lipid droplets in the cytoplasm is a specific marker of quiescent stellate cells, little is known about the mechanisms mediating their accumulation. Two recent studies by the same group of researchers have endeavoured to shed some light on this process. Kim and colleagues (Kim et al., 2009, 2010) have postulated that albumin (a protein that is endogenously expressed in PSCs and is co-localized with vitamin A in the lipid droplets) may play a role in lipid droplets formation. When the authors transfected activated PSCs (which had lost their lipid droplets) with expression plasmids for albumin, the cells exhibited a re-accumulation of lipid droplets that contained vitamin A (as confirmed by UV exposure). This was associated with increased resistance of the cells to the activating effects of the well known profibrogenic factor transforming growth factor beta (TGFβ). The authors have further shown that albumin is a downstream effector of the nuclear receptor peroxisome proliferator activated receptor γ (PPARγ) which is known to inhibit PSC activation (vide infra). While these studies provide interesting insights into the formation of vitamin A lipid droplets in PSCs, the mechanisms mediating their loss during PSC activation remain to be elucidated.

PSCs have the capacity to proliferate, to migrate and to synthesize and secrete proteins ECM proteins. Each of these functions is significantly stimulated during the activation process (see below). In addition to the production of ECM proteins, PSCs also produce matrix degrading enzymes (matrix metalloproteinases, MMPs) and their inhibitors (TIMPs, tissue inhibitor of metalloproteinases) (Phillips et al., 2003), suggesting that in health the cells may be responsible for the maintenance of normal ECM turnover in the pancreas. However, during pancreatic injury, when PSCs are activated, the balance between ECM production and ECM degradation is severely disturbed, leading to excessive ECM synthesis and eventually to the development of pathological fibrosis. With the availability of improved techniques for proteomic analyses based on mass spectrometry, three recent studies have assessed the differences in the proteomes of non-activated versus activated mouse (Paulo et al., 2011a), rat (Paulo et al., 2011b) and human (Wehr et al., 2011) PSCs. A detailed description of all differences is beyond the scope of this article, but it is interesting to note that numerous proteins were found to be differentially expressed in the two states, with proteins in the activated states being those related to the cell cytoskeleton, cell metabolism, motility, growth and invasion. (Table 1 summarizes the different characteristics of quiescent and activated PSCs).

**Table 1:** **Characteristics of quiescent and activated PSC phenotypes**.

	**Quiescent PSCs**	**Activated PSCs**
Vitamin A lipid droplets	Present	Absent
α Smooth muscle actin	Absent	Present
Proliferation	Limited	Increased
Migration	Limited	Increased
Extracellular matrix production	Limited	Increased
Matrix metalloproteinases (MMPs) and tissue inhibitors of matrix proteinases (TIMPs)	Complement of MMPs and TIMPs to maintain normal ECM turnover	Change in types of MMPs and TIMPs to facilitate ECM deposition
Production of cytokines	Limited	Increased (PDGF, TGFβ, CTGF, IL1, IL6, IL15)
Capacity for phagocytosis	Absent	Present
Proteomic analyses	Basal protein expression	Differential expression of proteins related to the cell cytoskeleton, cell metabolism, motility, growth and invasion

While the initial focus of PSC related research was on understanding the role of activated PSCs in fibrosis (discussed in detail later), more recent efforts have been directed toward other non-fibrogenic functions of quiescent cells. Accumulating evidence suggests that PSCs may function as (1) progenitor cells; (2) immune cells or (3) intermediary cells in cholecystokinin (CCK)-induced pancreatic digestive enzyme (exocrine) secretion. With regard to their possible progenitor function, Mato et al. (2009) isolated and expanded pancreatic cells from lactating rats using mitoxantrone (a drug that acts through multidrug transporter systems) selection. They have reported that the surviving, mitoxantrone-resistant cells showed a PSC-like morphology (fibroblast-like with vitamin A lipid droplets), expressed the stem cell marker ABCG2 transporter (ATP binding cassette G2 transporter) and were able to secrete insulin after cell differentiation. However, whether such a selected “drug resistant” population is representative of normal PSCs remains to be examined. In order to determine whether the cells are true progenitor cells, additional work is needed to assess whether PSCs express other stem cell markers and can transform (under physiological conditions) into other cell types.

In terms of an immune function, Shimizu et al. (2005) were the first to observe that PSCs could internalize necrotic acinar cells and apoptotic neutrophils, but this was associated with necrotic cell death of the PSCs themselves. These *in vitro* observations were supported by the authors' *in vivo* work using a mouse model of bile-duct ligation induced acute pancreatitis and a model of spontaneous chronic pancreatitis (WBN/Kob rats) in which they found that PSCs engulfed damaged parenchymal cells. Thus, the authors speculated that PSCs may exhibit a locally protective “innate” immune function to inhibit disease progression in early pancreatic injury. The role of PSCs in innate immunity is supported by the fact that the cells express Toll like receptors (TLR2, 3, 4, 5 and 9) which recognize foreign pathogen-associated molecular patterns (PAMPs) (Vonlaufen et al., 2007b; Masamune et al., 2008a; Nakamura et al., 2011). More recently, Shimizu and colleagues (2012) investigated whether PSCs may also have an “acquired” immune function by acting as antigen presenting cells. However, they found that rat PSCs did not express any antigen presenting cell markers such as MHC class II molecules or HLA-DR molecules. This finding differs from reports with HSCs which have been shown to process lipid antigens and present them to natural killer cells via CD1d. HSCs have also been shown to process protein antigens and present them to CD4 and CD8 positive T cells (Unanue, 2007; Winau et al., 2007). It is possible that the antigen-presenting capacity in HSCs develops because of the consistent exposure of the liver to foreign antigens from the gastrointestinal tract via the portal vein, whereas the pancreas (and PSCs) in comparison would be less likely to be exposed to the same load of exogenous antigens.

The question as to whether PSCs may play an intermediary role in CCK-induced digestive enzyme secretion arose from the known proximity of the PSCs to acinar cells *in situ* and the debate in the literature about the presence of functional CCK receptors on human acinar cells (in contrast to rat acinar cells where CCK receptors have been well identified). Two recent studies have convincingly demonstrated the presence of CCK receptors 1 and 2 on human PSCs (Berna et al., 2010; Phillips et al., 2010). Furthermore, the study by Phillips et al. (2010) has shown that PSCs respond to CCK by producing the neurotransmitter acetylcholine which can act on muscarinic receptors on acinar cells. Using a co-culture system of PSCs and acinar cells, the authors have also demonstrated an increase in amylase output by acinar cells in the presence of PSCs, which could be inhibited by the muscarinic receptor blocker atropine. These findings indicate that in humans, PSCs may play a significant intermediary role in regulating CCK-induced exocrine pancreatic secretion.

## Central role of activated PSCs in pancreatic fibrosis

*In vitro* and *in vivo* studies over the past 14 years (ever since the first descriptions of methods to isolate PSCs) have now convincingly demonstrated that when activated during pancreatic injury, PSCs play a critical role in the pathogenesis of pancreatic fibrosis.

## Activation of PSCs - *in vitro* studies

As noted earlier, transformation of PSCs from their quiescent to an activated state is a key event in fibrogenesis, resulting in excessive synthesis and deposition of ECM proteins. Specific molecules/factors and cellular pathways that mediate PSC activation were initially identified mostly by using cultured PSCs *in vitro*. The selection of putative activating factors for examination was based upon the knowledge that during the process of tissue injury, PSCs are likely to be exposed to factors such as: (1) alcohol and its metabolites acetaldehyde and fatty acid ethyl esters (FAEEs) [in view of the well known role of alcohol in pancreatitis (Apte et al., 2011)]; (2) endotoxin [given the known association of alcohol abuse and endotoxinaemia (Parlesak, 2005) and the correlation of circulating endotoxin levels with severity of pancreatitis (Windsor et al., 1993; Ammori et al., 1999)]; (3) growth factors and cytokines - transforming growth factor β (TGFβ), platelet derived growth factor (PDGF), tumour necrosis factor α (TNFα) and interleukins (IL), all of which are upregulated during pancreatic damage (Vonlaufen et al., 2007b); (4) oxidant stress (known to occur during both acute and chronic pancreatitis) (Uden et al., 1990; Casini et al., 2000); and (5) increased pancreatic pressure due to the “compartment syndrome” of chronic pancreatitis (Jalleh et al., 1991). To this list of activating factors have been added several others in recent years, on the basis of their overexpression in and/or association with chronic pancreatitis. These include hyperglycaemia [given that diabetes is a known complication of chronic pancreatitis (Nomiyama et al., 2007)], the endothelial cell derived vasoconstrictor endothelin-1 (Jonitz et al., 2009), cyclooxygenase 2 (COX-2, the inducible form of the rate limiting enzyme that converts arachidonic acid to prostaglandin) (Aoki et al., 2007), galectin-1 (a beta-galactoside binding lectin) (Masamune et al., 2006a) and the haemostatic protein fibrinogen (Masamune et al., 2009).

PSC activation in response to the above factors has generally been assessed using one or more of a number of “activation” parameters such as cell proliferation, αSMA expression, ECM protein synthesis, matrix degradation via the production of matrix metalloproteinases, loss of vitamin A stores, cell migration, cytokine release and contractility. Alcohol (ethanol) itself directly activates PSCs most likely due to the oxidative metabolism of ethanol to acetaldehyde via the enzyme alcohol dehydrogenase (ADH, known to be active in PSCs), and the subsequent generation of oxidant stress within the cell (Apte et al., 2000). Interestingly, ethanol upregulates PDGF-induced NADPH oxidase activity within PSCs (Hu et al., 2007), supporting the concept that reactive oxygen species (ROS) generated within PSCs play a role in PSC activation. It is noteworthy that ethanol can activate PSCs from their quiescent state and does not require the cells to be pre-activated to exert its stimulatory effects (Apte et al., 2000), suggesting that *in vivo*, PSC activation may occur early during chronic alcohol intake even in the absence of necroinflammation. This activation may then be perpetuated further during ethanol-induced necroinflammatory episodes leading to the development of fibrosis. Ethanol also inhibits PSC apoptosis (as assessed by the standard apoptosis indices Annexin V staining, TUNEL staining and caspase 3 and 9 activities) thereby facilitating cell survival (Vonlaufen et al., 2011). Furthermore, ethanol enhances the inhibitory effect of endotoxin lipopolysaccharide (LPS) on PSC apoptosis, suggesting that these two factors may exert synergistic effects on PSCs which promote cell activation and survival, thereby promoting pancreatic fibrosis. In contrast to the effects of the oxidative ethanol metabolite acetaldehyde on PSCs, the non-oxidative ethanol metabolites (FAEEs) have not yet been reported to activate PSCs. However, one of the FAEEs, palmitic acid ethyl ester (PAEE), has been shown to stimulate specific signaling molecules within PSCs (see below) (Masamune et al., 2004).

With regard to cytokines, it is now well established that (a) PDGF is a potent proliferative and chemotactic factor for PSCs; (b) TGFβ and its downstream effector connective tissue growth factor (CTGF) stimulate the synthesis and secretion of ECM proteins (collagen, fibronectin, and laminin) by PSCs; TGFβ also induces matrix metalloproteinase 2 (MMP2) production by the cells [it is postulated that degradation of normal basement membrane collagen by MMP2, facilitates the deposition of abnormal (fibrillar) collagen, thereby promoting fibrosis]; (c) the proinflammatory cytokines TNFα, monocyte chemotactic protein (MCP-1) and IL1, IL6 and IL13 stimulate proliferation, αSMA expression and/or collagen synthesis in PSCs (Apte et al., 1999; Schneider et al., 2001; Mews et al., 2002; Michalski et al., 2007).

**Figure 3:**
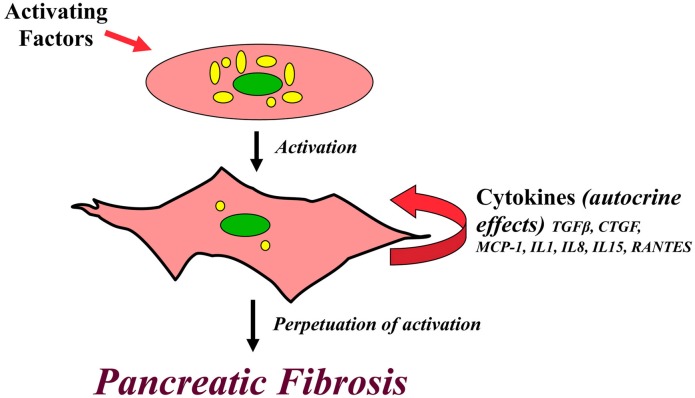
**Perpetuation of PSC activation.** A diagrammatic representation of the postulated pathway for a perpetually activated state for PSCs. Pancreatic stellate cells are activated via paracrine pathways by exogenous factors such as cytokines, oxidant stress, ethanol and its metabolites. Activated PSCs synthesize and secrete endogenous cytokines which influence PSC function via autocrine pathways. It is possible that this autocrine loop in activated PSCs perpetuates the activated state of the cell, even in the absence if the initial trigger factors, leading to excessive ECM production and eventually causing pancreatic fibrosis.

It is important to note that, in addition to responding to exogenous cytokines via paracrine pathways, PSCs themselves produce inflammatory mediators including TGFβ, CTGF, MCP-1, IL1, IL8, IL15 and RANTES (Regulated on Activation Normal T Cell Expressed and Secreted), all of which are capable of activating the cells via autocrine pathways (Andoh et al., 2000; Shek et al., 2002). The production of these endogenous cytokines can be stimulated by exogenous compounds such as ethanol, acetaldehyde, TGFβ and CTGF (Mews et al., 2002; Karger et al., 2008) and also by autocrine loops between certain cytokines in PSCs. For example, Aoki et al. (2006) have shown that IL-1β and IL6 produced by PSCs can each stimulate the autocrine secretion of TGFβ by the cells, and vice versa. In contrast IL13 (a Th2 lymphokine) suppresses TGFβ secretion by rat PSCs, although it induces PSC proliferation (Shinozaki et al., 2010). However, the relevance to human pancreatic fibrosis of the IL13 induced suppression of TGFβ secretion by rat PSCs is difficult to assess since the IL13 receptor system has not been detected in human pancreatitis specimens. Nonetheless, the ability of PSCs to be activated via autocrine pathways suggests that once activated, PSCs are capable of being in a perpetually activated state even in the absence of the initial trigger factors (Figure 3). This phenomenon may represent one of the mechanisms responsible for progression of chronic pancreatitis despite the cessation of the initial insult, for example alcohol and/or acute flare.

Oxidant stress (produced by exposure of cells to a pro-oxidant complex such as iron sulphate/ascorbic acid or hydrogen peroxide) activates PSCs and this activation is prevented by the antioxidant α-tocopherol (vitamin E) (Apte et al., 2000; Kikuta et al., 2004, 2006). PSCs have also been shown to generate ROS within the cell (Apte et al., 2000). Interestingly, (Masamune et al., 2008b) have demonstrated that PSCs express NADPH oxidase (an enzyme that is primarily found in phagocytic cells such as neutrophils and macrophages) to generate intracellular ROS, which, in turn, mediate activation of PSCs.

As noted earlier, pancreatic tissue pressure is elevated in chronic pancreatitis compared to normal pancreas. Asaumi et al. (2007) have reported activation of PSCs upon exposure to high pressure conditions (80 mmHg) produced using helium gas and a sealed pressure loading apparatus into which culture flasks bearing PSCs were placed. This activating effect was prevented by anti-oxidants such as N-acetyl cysteine (NAC) and epigallocatechin gallate (a green tea polyphenol), suggesting that it was mediated by intracellular oxidant stress.

Other factors that have recently been reported to induce PSC activation (as assessed by proliferation, migration, collagen production, αSMA expression or cytokine expression) include hyperglycaemia, endothelin 1, COX-2, galectin 1 and fibrinogen.

## Signaling pathways in PSCs

Having established the functional responses of PSCs to exogenous and endogenous factors, the logical next step for researchers in the field was to identify the intracellular signaling pathways mediating these responses, with the ultimate aim of developing approaches to target specific signaling molecules so as to interrupt PSC activation and inhibit abnormal fibrogenesis.

The activating effects of ethanol, acetaldehyde and oxidant stress on PSCs are mediated by activation of the mitogen activated protein kinase (MAPK) pathway (extracellular signal regulated kinase (ERK1/2), p38 kinase and c-jun amino terminal kinase (JNK), as well as the nuclear transcription factor AP-1 (Gukovskaya et al., 2002; McCarroll et al., 2003a,b). The non-oxidative metabolite of ethanol, PAEE also activates the same pathways in human PSCs (Masamune et al., 2004). Ethanol and acetaldehyde also activate two signaling molecules upstream of the MAPK cascade, phosphatidylinositol 3 kinase (PI3K) and protein kinase C (PKC) (McCarroll et al., 2003a,b). Given the synergistic effects of ethanol and endotoxin on PSCs *in vitro* (noted above), the LPS signaling pathway has recently been examined in PSCs. The cells express the LPS receptor TLR4 (toll like receptor 4) as well as the adapter molecules CD14 and MD2 (Vonlaufen et al., 2007b). Interestingly, TLR4 expression is upregulated in PSCs upon exposure to LPS (Vonlaufen et al., 2007b). PSCs are also known express other toll-like receptors, TLR2, 3 and 5 and exposure of the cells to relevant TLR ligands activates the transcription factor NFκB (Masamune et al., 2008a). This finding is of interest because NFκB can induce anti-apoptotic proteins such as IAPs (inhibitor of apoptosis proteins) (Bhanot and Moller, 2009) and may provide an explanation for the LPS-induced inhibition of PSC apoptosis observed *in vitro*.

PDGF-induced PSC proliferation is mediated by ERK and JAK/STAT (Janus activated kinases/Signal induced activation of transcription) (Jaster et al., 2002; Masamune and Shimosegawa, 2009), while PDGF-induced migration is regulated by the PI3K pathway (McCarroll et al., 2004). There is significant cross-talk between PI3K and ERK in PSCs, so that modulation of one pathway is often associated with a change in the function of the other (McCarroll et al., 2004). Another signaling molecule which influences PSC migration is Indian hedgehog (IHH) (Shinozaki et al., 2008), a peptide belonging to the hedgehog protein family that is active in pancreas development, patterning and differentiation. PSCs express smoothened (Smo) and patched-1 (Ptch1) proteins which are essential components of the hedgehog receptor system. IHH – receptor binding leads to relocation of the transcriptional factor Gli-1 to the nucleus and results in chemotactic as well as chemokinetic migration of PSCs. This is associated with localization of membrane type I – matrix metalloproteinase (MT1-MMP) to the surface of PSCs, where it is thought to aid basement membrane degradation so as to facilitate cell movement. The profibrogenic growth factor TGFβ exerts its effect on PSCs via the intracellular signaling mediators SMAD2 and 3 (Ohnishi et al., 2004). TGFβ also exerts autocrine effect on PSCs whereby it induces its own mRNA expression; this process is regulated by the ERK pathway (Ohnishi et al., 2004).

The well-established association of PSC activation with the expression of the cytoskeletal protein αSMA, has prompted studies on the regulation of the actin cytoskeleton and PSC morphology. The small GTP protein Rho and its downstream effector Rho kinase regulate the actin cytoskeleton, stress fibre formation and alteration of cell shape during the PSC activation process (Masamune and Shimosegawa, 2009).

Intracellular calcium signaling, which is closely linked to the pathways mentioned above is modulated in PSCs in response to the binding of growth factors and cytokines to relevant receptors on the cell surface (Masamune and Shimosegawa, 2009). PSCs also respond to the extracellular nucleotides purines and pyrimidines (known to be involved in cell-cell communication of inflammatory signals after cell injury) via P2X and P2Y receptors (Hennigs et al., 2011). Activation of P2 receptors elicits robust intracellular Ca signaling known to mediate the fibrogenic function of activated PSCs (Hennigs et al., 2011).

Most recently, attention has turned toward microRNAs, the small non-coding RNAs that are implicated in many biological processes including cell differentiation, proliferation, apoptosis and tumorigenesis. Shen et al. (2012) have reported that miR-15b and miR-16 modulate rat PSC apoptosis by targeting the anti-apoptotic factor Bcl-2.

While the above discussion focuses on PSC activation pathways, factors and signaling molecules that inhibit PSC activation have also been examined in recent times. In this regard, it is now known that inhibition of MAPK (ERK, JNK and p38 kinase) signaling mediates the induction of quiescence of PSCs in response to retinol and its metabolites ATRA and 9-cisRA (McCarroll et al., 2003a,b). Curcumin, a polyphenol compound found in turmeric, decreases PDGF-induced PSC proliferation via inhibition of the ERK pathway; it also inhibits cytokine-induced PSC activation by inhibiting the MAPK pathway and by preventing activation of the transcription factor AP-1 (Masamune et al., 2006b). The peroxisome proliferator-activated receptor γ (PPAR γ, a ligand-activated transcription factor which controls cellular growth and differentiation) mediates the inhibitory effect of its ligand troglitazone on PDGF-induced and culture-induced activation of PSCs (Masamune et al., 2002; Shimizu et al., 2004).

## PSCs in chronic pancreatitis

The role of PSCs in chronic pancreatitis has been assessed predominantly via *ex vivo* studies using pancreatic sections from patients with chronic pancreatitis and *in vivo* studies using animal models of chronic pancreatitis.

### Human studies

**Figure 4:**
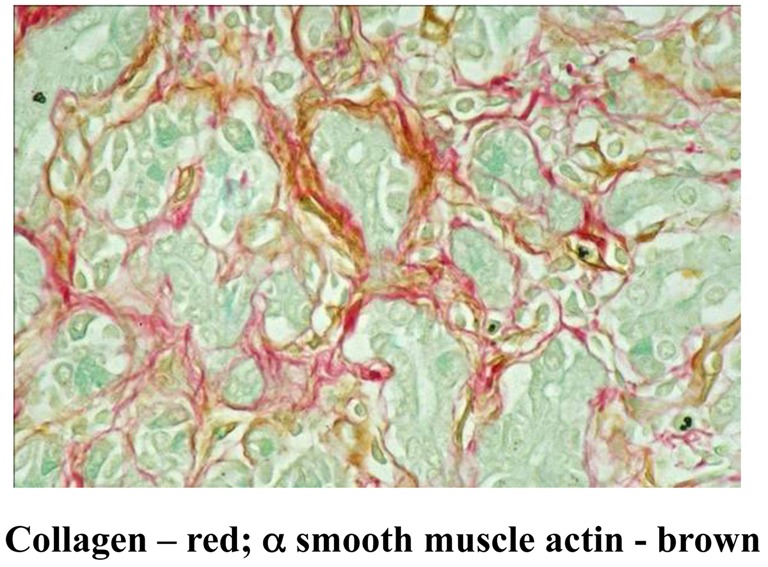
**Dual staining of a human chronic pancreatitis section immunostained for the PSC activation marker α smooth muscle actin (αSMA) and for collagen using Sirius Red.** The brown staining for αSMA is co-localized with the red staining for collagen indicating the presence of activated PSCs in fibrotic areas of the pancreas. *Reprinted with permission from Elsevier*.

Human chronic pancreatitis sections have mainly been examined using standard histological stains (H and E, Masson's trichrome for connective tissue and Sirius Red for collagen) and using immunohistochemistry for the presence of specific proteins. These studies have clearly established that in the fibrotic pancreas, areas that stain positive for collagen also stain for alpha smooth muscle actin indicating the presence of activated PSCs (Figure 4). Moreover, using dual immunostaining for αSMA, it has been established that it is predominantly the activated PSCs that produce the collagen in the fibrotic areas (Haber et al., 1999).

The expression of growth factors known to activate PSCs is also increased in chronic pancreatitis. Pancreatic acinar cells adjacent to areas of fibrosis exhibit strong staining for TGFβ, while such staining is absent in acinar cells remote from bands of fibrosis (Haber et al., 1999), suggesting that TGFβ secreted by pancreatic acinar cells may have a paracrine effect on PSCs, leading to increased collagen synthesis by the cells. TGFβ staining is also evident in spindle shaped cells in the fibrotic bands. The expression of NGF, one of the stellate cell selective markers) is also increased in human chronic pancreatitis (Friess et al., 1999). Since NGF is expressed by neuronal cells as well as PSCs, it is possible that proliferating PSCs in fibrotic areas may contribute to the observed increase in NGF staining in this disease. Expression of the receptor for PDGF is also increased (at both mRNA and protein levels) in areas of fibrosis in chronic pancreatitis (Haber et al., 1999). Given that PDGF is a potent mitogenic and chemotactic factor for PSCs, increased PDGF receptor expression on the cells may be one of the mechanisms responsible for the increased numbers of PSCs observed in fibrotic areas.

Interestingly, there is evidence of increased oxidant stress in fibrotic areas of chronic pancreatitis as indicated by positive staining for 4-hydroxynonenal (4HNE, a lipid peroxidation product) (Casini et al., 2000). This finding is highly relevant because PSCs are known to be activated in response to oxidant stress (as noted earlier).

### Animal models

The human studies described above were important in terms of confirming the association of activated PSCs with pancreatic fibrosis in chronic pancreatitis. However, being cross-sectional studies, they did not allow examination of chronological events in the development of pancreatic fibrosis. This limitation was overcome by studies with animal models (predominantly rodent models) of pancreatic fibrosis. Fibrosis has been produced in rodents via various approaches. Rat models described in the literature include: (1) trinitrobenzene sulfonic acid (TNBS) injection into the pancreatic duct (Haber et al., 1999); (2) intravenous injection of an organotin compound dubutyltin chloride (DBTC) (Emmrich et al., 2000); (3) spontaneous chronic pancreatitis in WBN/Kob rats (Ohashi et al., 1990); (4) severe hyperstimulation obstructive pancreatitis (SHOP), involving intraperitoneal (IP) injections of supramaximal doses of caerulein (a synthetic analogue of CCK, a major pancreatic secretagogue) + bile-pancreatic duct ligation (Murayama et al., 1999); (5) repeated IP injections of a superoxide dismutase inhibitor (Matsumura et al., 2001); (6) intragastric high dose alcohol administration + repeated caerulein injections (Tsukamoto et al., 1988; Uesugi et al., 2004); (7) chronic alcohol administration (liquid diet) with repeated cyclosporin and caerulein injections (Gukovsky et al., 2008) and (8) chronic alcohol administration with repeated endotoxin LPS, injections (Vonlaufen et al., 2007b). Mouse models of pancreatic fibrosis include: (i) transgenic mice overexpressing TGFβ or the EGF receptor ligand heparin binding epidermal growth factor-like growth factor (HB-EGF) (Blaine et al., 2009); (ii) repetitive pancreatic injury induced by repeated injections of supramaximal caerulein (Neuschwander-Tetri et al., 2000); (iii) transgenic mice overexpressing IL-1β (Marrache et al., 2008b).

The overall results from animal studies to date support the concept that PSCs are activated early in the course of the injury, most likely due to paracrine effects of factors (cytokines and ROS) produced by injured acinar cells and/or inflammatory cells during the acute phase of the injury. Activated PSCs are the major source of collagen in fibrotic areas (confirming findings from in human studies). As with human chronic pancreatitis, expression of factors known to activate PSCs are all reported to be upregulated in experimental pancreatic fibrosis including PDGF and its receptor, TGFβ and two TGFβ regulated genes SM22α and Cygb/STAP, and oxidant stress.

Although the above models have provided useful data, caution needs to be exercised in assessing their direct clinical relevance, since most have involved relatively non-physiological methods (e.g., injections of toxin into the pancreatic duct, administration of supraphysiological levels of caerulein or interventions such as bile duct ligation), to produce pancreatic damage. However, there is one rat model produced by chronic alcohol administration and repeated endotoxin exposure (Vonlaufen et al., 2007a,b) that is based on a well recognized clinical phenomenon, namely endotoxinaemia (secondary to increased gut mucosal permeability) in alcoholics (Bode et al., 1993; Parlesak, 2005). Thus, the alcohol feeding, LPS challenge model possibly represents the most physiologically relevant model of chronic alcoholic pancreatitis described to date.

### Source of activated PSCs in the fibrotic pancreas - evidence from animal models

The significant increase in PSC numbers observed during pancreatic injury has raised the question as to whether these increased numbers are made up largely of resident “pancreatic” PSCs or whether migratory cells homing to the pancreas from extra-pancreatic sources such as the bone marrow (in response to chemotactic signals from the injured organ) also contribute to the PSC population. Two recent studies have used a gender mismatch and chimeric approach whereby green fluorescent protein (GFP) labeled bone marrow derived cells (BMDC) obtained from GFP transgenic male donor mice were transplanted into lethally irradiated wild type female rodents (Marrache et al., 2008a,b; Sparmann et al., 2010). Pancreatic injury was then induced in recipient mice either by repeated injections of caerulein or using the chemical toxin dibutyltin chloride (DBTC). Both studies showed that a small proportion (5–18%) of the proliferative PSCs in the pancreas could be bone marrow derived, but additional studies with different models of pancreatic fibrosis need to be performed to fully characterize the contribution of bone marrow derived PSCs to progression (or repair) of pancreatic injury.

## Fate of activated PSCs and reversal of pancreatic fibrosis

As the processes of PSC activation are becoming increasingly clear, the fate of activated PSCs is also attracting increasing attention. Three possibilities that have been considered include: (1) reversion to quiescence; (2) apoptosis; and (3) senescence. Partial reversion to quiescence has been described *in vitro* upon exposure to retinol and its metabolites, albumin or culture on matrigel (a basement membrane like matrix) (McCarroll et al., 2003b; Kim et al., 2009), however, there is no *in vivo* evidence yet to support these findings. On the other hand, apoptosis of PSCs has been well described *in vitro* and also recently *in vivo* using the alcohol-fed endotoxin challenged model of chronic pancreatitis. Vonlaufen et al. (2011) have demonstrated increased apoptosis of activated PSCs *in vivo* upon withdrawal of alcohol in this model. In terms of cell senescence, a very recent paper by Fitzner and colleagues (2012) has reported that PSCs in long-term culture (6 weeks) express the senescence marker senescence associated b-galactosidase (SA-βGal) and are highly susceptible to immune cell-mediated cytotoxicity. Furthermore, the authors report that in a rat model of DBTC-induced pancreatitis, PSCs not only express activation markers, but also senescence markers leading them to speculate that inflammation, PSC activation and senescence are timely coupled processes in the injured pancreas. However, this study did not assess PSC apoptosis. Indeed, the relative contributions of the processes of apoptosis versus senescence versus reversion to quiescence in the removal of activated PSCs after pancreatic injury remain to be clarified.

Regardless of the eventual fate of activated PSCs, advances in our knowledge of the processes of PSC activation have helped underpin evidence-based rationales for the development of potentially useful anti-fibrotic therapies *in vivo* (albeit only in experimental models so far). Several treatments/approaches have been reported to prevent/retard fibrosis in animal models, including: (i) Antioxidants - vitamin E (the subclass tocotrienol has been shown to induce PSC death via apoptosis and autophagy) (Gomez et al., 2004; Vaquero et al., 2007), oxypurinol and allopurinol, both xanthine oxidase inhibitors (Pereda et al., 2004; Tasci et al., 2007), ellagic acid, a plant derived polyphenol with antioxidant, anti-inflammatory and anti-fibrosis activities (Suzuki et al., 2009), and salvianolic acid, a herbal medicine with free radical scavenging properties (Lu et al., 2009); (ii) TGFβ suppression - using TGFβ neutralizing antibodies (Menke et al., 1997), a herbal medicine Saiko-keishi-to (Su et al., 2001) or a plant alkaloid halofuginone which inhibits downstream Smad3 phosphorylation (Zion et al., 2009); (iii) TNFα inhibition - using a TNFα antibody (Hughes et al., 1996), soluble TNFα receptors or an inhibitor of TNFα production pentoxifylline (Pereda et al., 2004); (iv) anti-inflammatory agents - protease inhibitors such as camostat mesilate which inhibit proinflammatory cytokine production by monocytes (Gibo et al., 2005) and the synthetic carboxamide derivative IS-741 which suppresses macrophage infiltration into the pancreas, with a consequent decrease in *in vivo* PSC activation (Kaku et al., 2007); and (v) modulation of signaling molecules using the PPARγ ligand troglitazone (Shimizu et al., 2004).

In terms of alcoholic pancreatic fibrosis, withdrawal of alcohol from the diet after established early pancreatitis, has been shown to result in complete reversal of pancreatic fibrosis (Vonlaufen et al., 2011). The authors have attributed this effect to the fact that the alcohol-induced inhibition of PSC apoptosis (described earlier) is removed in the absence of alcohol, thereby enabling the loss of activated PSCs through cell death and interrupting the fibrogenic process. These observations provide a strong experimental basis for the advocacy of abstinence in patients with alcoholic pancreatitis in a bid to prevent disease progression.

## PSCs in pancreatic cancer

Pancreatic ductal adenocarcinomas are characterized by an abundant stromal/desmoplastic reaction which, up until recently had received little attention in terms of its possible role in the pathogenesis of the disease. It was the emerging evidence of the central role of PSCs in the fibrosis of chronic pancreatitis that stimulated researchers to investigate whether the same cells were responsible for the production of the stroma of pancreatic cancer, and if so, whether PSCs interacted with cancer cells to influence disease progression (Apte and Wilson, 2012). Of particular relevance in this regard were the known increased risk of pancreatic cancer in patients with chronic pancreatitis (Raimondi et al., 2010) and the commonalities in gene expression between the stromal compartments of chronic pancreatitis and pancreatic cancer (Binkley et al., 2004).

**Figure 5:**
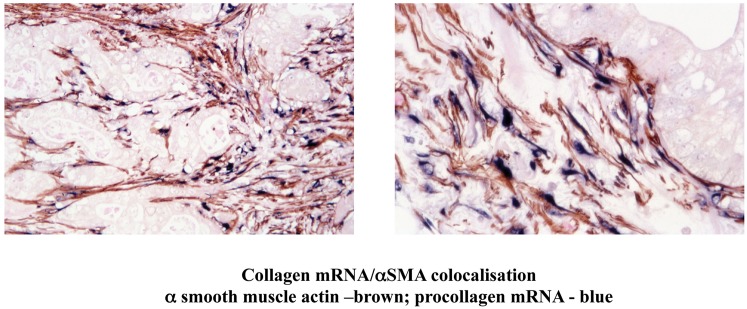
**Dual staining of a human pancreatic cancer section for α smooth muscle actin (αSMA) and mRNA for collagen.** The Figure depicts low and high power views of a pancreatic cancer section immunostained for αSMA and for collagen mRNA using *in situ* hybridisation. The brown staining for αSMA is co-localized with the blue staining for collagen mRNA. Importantly, both stains are restricted to the stromal areas of the section, with no staining of the tumour elements. The findings indicate that activated PSCs are the predominant source of collagen in the stroma of pancreatic cancer. *Reprinted with permission from Wolters Kluwer Health*.

Histological and immunohistochemical studies of human pancreatic cancer sections have shown that activated PSCs are present in the desmoplastic areas of pancreatic cancer (Apte et al., 2004). Furthermore, dual staining for activated PSCs (αSMA) and for collagen mRNA (*in situ* hybridisation) has established that the predominant cells responsible for producing the fibrosis in pancreatic cancer are PSCs (Figure 5) (Apte et al., 2004).

The possibility of a close interaction between PSCs and pancreatic cancer cells has been examined *in vitro* (using co-cultures of PSCs and pancreatic cancer cell lines and/or exposure of one cell type to conditioned medium from the other) as well as *in vivo* (using subcutaneous, orthotopic and transgenic mouse models of pancreatic cancer) (Apte and Wilson, 2012).

### *In vitro* studies

**Figure 6:**
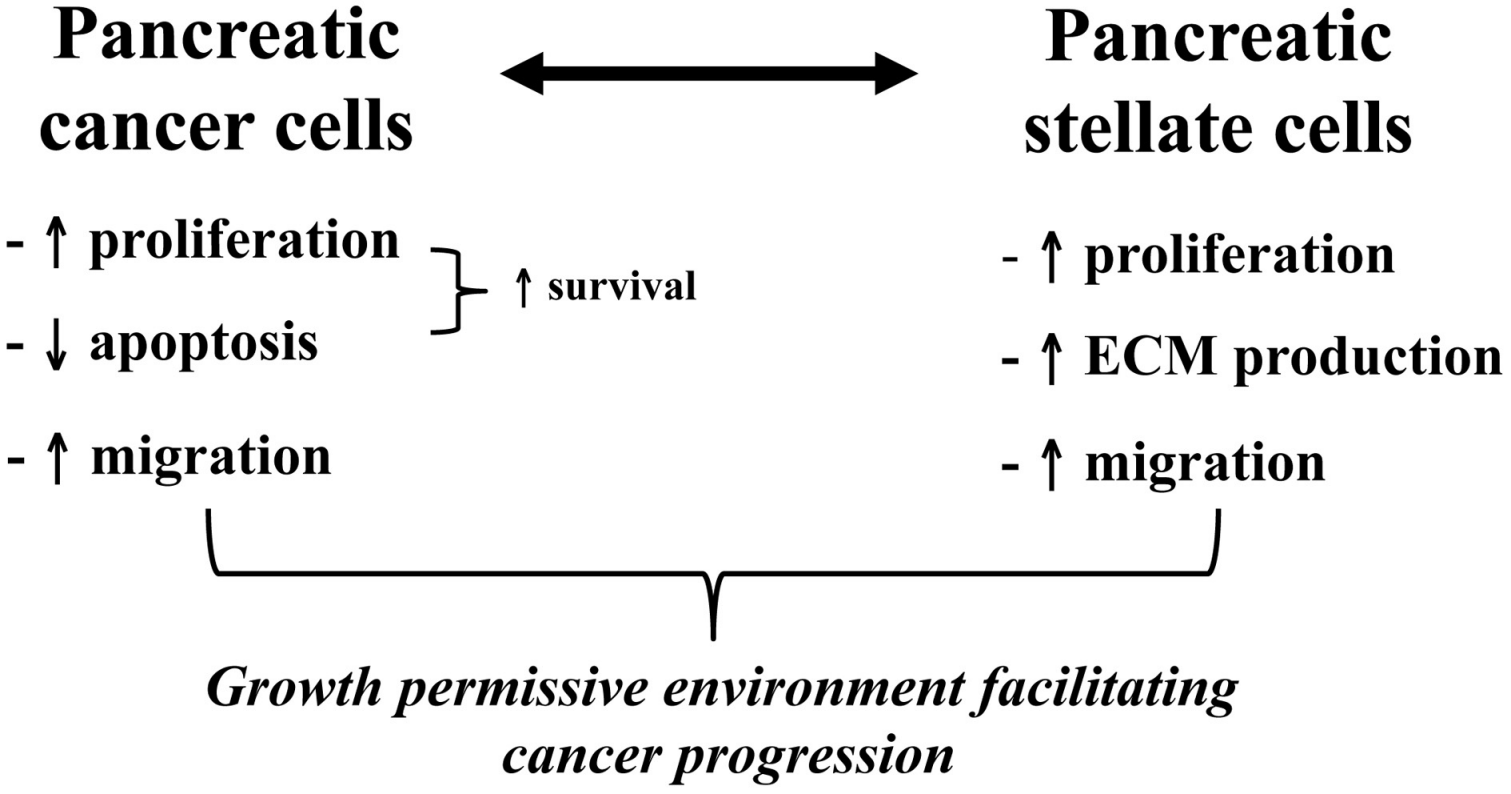
**Bidirectional interactions between pancreatic cancer cells and PSCs.** The diagram outlines the effects of pancreatic cancer cells on PSCs and vice versa. The observed interaction between these two cell types facilitates local tumour growth as well as regional and distant metastasis of pancreatic cancer.

Exposure of PSCs to cancer cells (either directly or via conditioned media) results in activation of PSCs (increased proliferation, ECM synthesis, and migration) (Apte and Wilson, 2012). In turn, PSCs stimulate cancer cell proliferation but inhibit cancer cell apoptosis thereby effectively enhancing the survival of cancer cells and induce cancer cell migration. The PSC-induced cancer cell migration is associated with epithelial-mesenchymal transition in cancer cells as indicated by decreased expression of epithelial markers such as E-cadherin and increased expression of mesenchymal markers such as vimentin and Snail in cancer cells (Kikuta et al., 2010). It is possible that this PSC-induced epithelial-mesenchymal transition in cancer cells facilitates the migration of these cells. Recently, a study by Ikenaga et al (Ikenaga et al., 2010) has reported that a subset of PSCs that overexpress CD10 (a cell membrane associated MMP) induce cancer cell invasion and proliferation significantly more than CD10 negative PSCs, suggesting that functional heterogeneity of PSCs may influence their effects on tumour progression. Overall, the above observations suggest that pancreatic cancer cells recruit host PSCs to their immediate vicinity and that PSCs reciprocate by facilitating cancer cell growth as well as local invasion (Figure 6). Most recently, it has been reported that PSCs increase the stem cell phenotype of cancer cells, as assessed by increased expression of stem cell markers such as nestin, ABCG2, and LIN28 in cancer cells upon co-culture with PSCs (Hamada et al., 2012). These findings have implications for the possible resistance to treatment of a cancer stem cell niche which then facilitates recurrence of tumour.

The cancer cell induced increase in ECM synthesis by PSCs is thought to be mediated by TGFβ1 and fibroblast growth factor 2 (FGF2), while PSC proliferation is likely mediated by PDGF. Recent studies have also implicated cycloxygenase 2 (COX-2, the inducible form of cycloxygenases, enzymes involved in conversion of arachidonic acid to prostaglandin) (Hughes et al., 2005) and trefoil factor 1 (TFF1) (Arumugam et al., 2011) (a stable secretory protein that is upregulated in pancreatic cancer but not expressed in the normal pancreas) in PSC proliferation in response to cancer cell secretions. COX-2 is upregulated in PSCs exposed to the pancreatic cancer cell line PANC1 and inhibition of COX-2 prevents PANC1 induced PSC proliferation. ERK1/2 has been identified as the signaling pathway regulating cancer cell-induced PSC proliferation (Hughes et al., 2004).

The possible factors mediating the effects of PSCs on cancer cells remain to be characterized. However, PSC-induced proliferation of cancer cells is thought to be mediated, at least in part, by PDGF (Xu et al., 2010). Other candidate factors in PSC secretions that require further study as possible mediators include the growth factors insulin-like growth factor (IGF), EGF, hepatocyte growth factor (HGF), TGFß and other proinflammatory cytokines.

### *In vivo* studies

In order to obtain *in vivo* evidence to support the stromal-tumour interactions observed *in vitro*, scientists have turned to murine xenograft or transgenic models. Using an immunocompromised mouse model of pancreatic cancer produced by subcutaneous injection of either a suspension of pancreatic cancer cell cells alone or an admixture of cancer cells and PSCs, Bachem et al. (2005) have shown a significantly increased rate of tumour growth in the latter group. The larger tumours in the mice injected with cancer cells + PSCs were due not only to the expected PSC-mediated fibrosis but also to proliferation of tumour cells themselves, suggesting that the presence of PSCs stimulated cancer cell growth.

One of the drawbacks of subcutaneous xenografts is the absence of the natural tumour microenvironment. Therefore, orthotopic models which involve injection/implantation of cancer cells directly into the organ of interest are a preferred option. In these models, tumours develop in a relevant anatomical location, so that the implanted cancer cells are exposed to the same microenvironment as may be expected in human cancer. In addition, orthotopic tumours have the capacity to metastasise thus allowing studies of tumour progression. Early studies by Lohr et al. (2001) reported that orthotopic injections of pancreatic cancer cells (PANC-1 cell line) transfected with TGFβ1 cDNA resulted in the induction of an extensive stromal reaction around the pancreatic tumour. Although not specifically studied at the time, this stromal reaction was most likely via the TGFβ-induced activation of stromal cells/fibroblasts/ in the host (mouse) pancreas.

More recently, orthotopic models of pancreatic cancer have been described wherein human pancreatic cancer cells (MiaPaCa-2, AsPC-1) with or without human pancreatic stellate cells (hPSCs) were injected directly into the mouse pancreas (Vonlaufen et al., 2008; Xu et al., 2010). In the presence of hPSCs, local tumour growth, and importantly, regional and distant metastasis were significantly enhanced. Tumours produced by the mixture of cancer cells and PSCs exhibited bands of fibrosis (resembling desmoplasia) and the presence of αSMA positive activated PSCs as well as increased proliferation and decreased apoptosis of cancer cells. These data concur well with the interactions between PSCs and cancer cells observed *in vitro* and strongly support an active role for PSCs in cancer progression (increased local growth and distant metastasis).

Neo-angiogenesis is a well recognized event in malignant tumours and is thought to be a major factor influencing cancer metastasis. PSCs significantly enhance tumour angiogenesis as indicated by upregulation of the endothelial cell marker CD31 in orthotopic tumours produced by cancer cell + PSCs compared to tumours produced by injection of cancer cells alone (Xu et al., 2010). These *in vivo* findings are supported by *in vitro* observations that PSCs stimulate tube formation of human microvascular endothelial cells, an effect that is mediated by vascular endothelial growth factor (VEGF) secreted by PSCs (Xu et al., 2010).

The process of angiogenesis in human pancreatic cancers may be somewhat more complex than that in mouse models. Studies with human pancreatic cancer sections indicate that neo-angiogenesis is limited to the invading front of the tumour while the central areas of the tumour have few patent blood vessels and are relatively hypoxic (Erkan et al., 2009). In an attempt to address this issue, Erkan et al. (2009) assessed the effects of hypoxia on the interactions of PSCs with endothelial cells. Using co-cultures of the two cell types, they found that the VEGF-mediated proliferative effect of PSCs on endothelial cells observed under normoxic conditions were dampened under hypoxic conditions. At the same time however, hypoxia significantly increased PSC activation and ECM synthesis. Further studies are needed to clarify the relative importance of new blood vessel formation versus tumour hypoxia in terms of the influence of PSCs on cancer behavior.

One of the intriguing features of PSC biology reported recently, is the ability of the cells migrate through an endothelial layer *in vitro*, suggesting that PSCs have the capacity to intravasate/extravasate to and from blood vessels *in vivo* (Xu et al., 2010). In the presence of cancer cells transendothelial migration of PSCs is further stimulated, an effect which may be mediated by PDGF in cancer cell secretions (Xu et al., 2010). More interestingly, it has now been shown, using a gender mismatch approach, that PSCs from the primary tumour (produced by implantation of female pancreatic cancer cells + male PSCs into the pancreas of female mice) can be detected in distant metastatic sites as y chromosome positive cells using fluorescent *in situ* hybridization. These findings suggest that PSCs can travel to distant metastatic sites (possibly with cancer cells) where they likely facilitate the seeding, survival, and growth of cancer cells. Indeed, similar observations have now been reported in a model of lung cancer (Duda et al., 2010). These findings challenge the long held concept that metastasis is the sole preserve of cancer cells.

The above studies provide convincing evidence of an active role of PSCs in pancreatic cancer progression. It is now also acknowledged that PSCs (via the production of dense stroma) may play a role in the well documented resistance of pancreatic cancer to chemotherapy and radiotherapy (Hanahan and Weinberg, 2011). In this regard, Olive et al. (2009) have shown in an orthotopic model of pancreatic cancer, that gemcitabine (a widely used chemotherapeutic agent for pancreatic cancer) is sequestered in the stromal area of pancreatic cancer, thereby limiting the availability of drug to cancer cells and providing a possible explanation for the chemoresistance of the disease. In addition, Mantoni and colleagues (2011) have reported that PSCs protect cancer cells from radiation via a ß1-integrin dependent pathway.

Given the accumulating evidence of the influence of PSCs on pancreatic cancer behavior, it is logical that the stroma is now seen as an important alternative therapeutic target to improve the outcome of this disease. Several recent studies have reported encouraging findings with such approaches in pre-clinical models. In a transgenic mouse model of pancreatic cancer, Olive et al. (2009) have shown that inhibition of the Sonic hedgehog pathway in PSCs (achieved by using vitamin A containing liposomes to specifically target stellate cells) transiently decreased PSC activation resulting in stromal depletion and increased accumulation of gemcitabine in cancer cells leading to cancer cell destruction. Von Hoff et al. (2011) have observed stromal depletion upon treatment of subcutaneous xenografts in mice with nanoparticle albumin bound paclitaxel, while Froeling and colleagues (2001) have reported decreased tumour growth in a transgenic mouse model of pancreatic cancer treated with the PSC inhibitor all trans retinoic acid (ATRA). It is anticipated that ongoing research will also target the signaling pathways/molecules that mediate PSC-cancer cell interactions so as to inhibit the facilitatory effects of PSCs on cancer progression.

To conclude, over the past two decades there has been a steep rise in our understanding of pancreatic fibrogenesis and the central role of PSCs in this process. It is also clear that PSCs have functions over and beyond the regulation of pathologic fibrosis in the pancreas, with the cells likely playing important roles in health as immune and/or progenitor cells and as intermediary cells in digestive enzyme secretion (at least in humans). Improved understanding of PSC biology will underpin the development of novel therapies in the future for the treatment of chronic pancreatitis and pancreatic cancer.

### Conflict of interest statement

The authors declare that the research was conducted in the absence of any commercial or financial relationships that could be construed as a potential conflict of interest.

## Abbreviations

PSCs: pancreatic stellate cells
PDGF: platelet derived growth factor
TGFb: transforming growth factor beta
HGF: hepatocyte growth factor
VEGF: vascular endothelial growth factor
IL: interleukin
NFkB: nuclear factor kappa B
AP-1: activator protein 1
αSMA: alpha smooth muscle actin
TLR: toll like receptor.
